# Risk factors associated with liver‐related complications in diabetes: There may be more than meets the eye

**DOI:** 10.1002/ueg2.12514

**Published:** 2023-12-19

**Authors:** Lukas Otero‐Sanchez, Christophe Moreno

**Affiliations:** ^1^ Department of Gastroenterology, Hepatopancreatology and Digestive Oncology Hôpital Universitaire de Bruxelles Université Libre de Bruxelles Brussels Belgium; ^2^ Laboratory of Experimental Gastroenterology Université Libre de Bruxelles Brussels Belgium

**Keywords:** alcohol consumption, chronic liver disease, metabolic dysfunction‐associated fatty liver disease, screening strategy, type 2 diabetes

Over the past 2 decades, there has been a growing scientific interest regarding the relationship between diabetes and liver‐related complications. To date, PubMed has indexed 44,334 papers encompassing the terms ‘diabetes’ and ‘liver disease’.^1^ Notably, over a quarter of these papers specifically focus on the interplay between diabetes and metabolic dysfunction‐associated fatty liver disease (MASLD).

Diabetes, mostly type 2 diabetes (T2DM), and MASLD are two pathologic conditions that frequently coexist and synergically impact the risk of liver and non‐liver related outcomes. Obesity and insulin resistance are key pathogenic factors shared by both MASLD and T2DM.

Patients with T2DM have emerged as the dysmetabolic subpopulation exhibiting the highest risk for liver fibrosis development and progression, with a global prevalence of advanced fibrosis ranging between 14% and 17%.^2^, ^3^, ^4^ Given the high prevalence of advanced fibrosis and the limited accuracy of non‐invasive blood tests (e.g., fibrosis‐4 index (FIB‐4)) in predicting advanced fibrosis and liver‐related complications in this population, it is surprising that vibration‐controlled transient elastography (VCTE) is not readily recommended as a first‐line screening strategy.^5^ More specifically for individuals at higher risk, including those who are obese, older, and those on insulin therapy.^3^, ^6^

However, it should be noted that the majority of studies assessing risk factors associated with liver‐related complications in the diabetic population are primarily focused on MAFLD, which per se exclude those patients with excessive alcohol consumption. The new classification of steatotic liver disease provides the unique opportunity to include more phenotypically diverse patients, encompassing those with chronic heavy alcohol consumption. This will allow for a clear dissection of the attributable risk fraction for liver outcomes conferred by each risk factor, both cardiometabolic and alcohol‐related. However, this inclusivity probably should not be pursued with the aim of homogenizing populations but rather to provide a clearer understanding of which risk factors confer the greatest increment in the risk of developing liver complications.

In this issue of UEG *Journal*, Michel et al. report advanced fibrosis prevalence and related‐risk factors in a prospective cohort of patients with T2DM recruited from two German primary care centers.^7^ Using VCTE screening, the authors report a significant high prevalence of advanced fibrosis (18.3%) and cirrhosis (12%). Furthermore, authors report harmful alcohol consumption (AUDIT score ≥8) as the primary independent risk factor associated with the diagnosis of liver cirrhosis (20% of cirrhosis cases had harmful alcohol consumption vs. 8% in for the whole cohort). This study highlights the high prevalence of advanced fibrosis in adults with T2DM, even in primary care. Moreover, it underscores the role of chronic alcohol consumption in the increased risk of developing liver cirrhosis in this specific population.

In this regard, a recent study by Mallet et al., evaluated risk factors associated with liver disease progression to hepatocellular carcinoma and/or decompensated cirrhosis in 52,066 hospitalized patients with T2DM.^8^ The authors reported that alcohol use disorders accounted for more than half of the attributable risk fraction for liver‐related complications, while features of the metabolic syndrome (i.e., obesity) contributed to less than 10%. Another cohort study, which followed 1068 diabetic patients for 15 years, suggests that a more pronounced insulin‐resistant profile and excessive alcohol consumption at diabetes diagnosis, rather than an elevated body mass index, were the main risk factors associated with subsequent liver‐related events.^9^ Considering the pivotal role of alcohol consumption as a risk factor for liver complications in both diabetic and non‐diabetic dysmetabolic populations, there is a pressing need to evaluate precise scales for quantifying alcohol consumption. In line with this, a recent study revealed that 29% of patients classified initially as MAFLD had relevant alcohol intake when assessed using objective alcohol markers.^10^ This will not only allow for a more accurate assessment of alcohol effects but also facilitates risk stratification and prioritization of interventions, especially in dysmetabolic patients.

Current joint guidelines from the American Association for the Study of Liver Diseases and the European Association for the Study of the Liver advocate for utilizing FIB‐4 as a primary risk assessment tool for advanced liver fibrosis, even in adults with T2DM.^11^, ^12^ However, considering the elevated prevalence of advanced fibrosis and the low negative predictive value of FIB‐4 in this specific population, there is an urgent need to assess the cost‐effectiveness of integrating VCTE as the primary risk assessment strategy in patients with T2DM. Furthermore, considering the significant impact of chronic alcohol consumption on the increased risk of developing advanced fibrosis in this population, it would be beneficial to standardize the screening for alcohol use disorders among T2DM. Integrating this into screening policies could effectively identify those at the highest risk of liver‐related complications.

## AUTHOR CONTRIBUTIONS

LOS, and CM designed the editorial. All authors critically revised the manuscript and approved the final version to be published.

## CONFLICT OF INTEREST STATEMENT

Lukas Otero‐Sanchez has nothing to disclose. Christophe Moreno was paid as speaker or adviser from Echosens, Surrozen, Intercept, and Gildead Sciences pharmaceutical companies. He is consultant for Julius clinical. He received research Grant from Gilead Sciences.

## FUNDING INFORMATION

Fonds Erasme

## Data Availability

Data sharing not applicable to this article as no datasets were generated or analyzed during the current study.
